# Hydroxyurea-Loaded Albumin Nanoparticles: Preparation, Characterization, and In Vitro Studies

**DOI:** 10.3390/pharmaceutics11080410

**Published:** 2019-08-12

**Authors:** Yerkeblan Tazhbayev, Olzhas Mukashev, Meiram Burkeev, Jörg Kreuter

**Affiliations:** 1 Chemical Materials Science and Nanochemistry Laboratory, Buketov Karaganda State University, Karaganda 100026, Kazakhstan; 2 Institute of Pharmaceutical Technology, Goethe University Frankfurt am Main, D-60438 Frankfurt am Main, Germany; 3 I.M. Sechenov First Moscow State Medical University, 119991 Moscow, Russia

**Keywords:** human serum albumin, nanoparticles, hydroxyurea, cancer

## Abstract

Human serum albumin nanoparticles (HSA-NPs) have been widely used as drug delivery systems. In most cases, HSA-NPs are formed by the method of desolvation in the presence of glutaraldehyde as a crosslinking agent. In the present study, we showed the possibility of crosslinking human serum albumin (HSA) molecules with natural agents, urea, and cysteine at the nanoparticle level under mild conditions (at room temperature of 20–25 °C). Optimal concentrations of the interacting components (HSA, urea, and cysteine) were found to produce nanoparticles with optimal physico-chemical parameters (particle size, polydispersity, zeta potential, yield, etc.) for application as drug carriers. We used hydroxyurea (HU), a simple organic compound currently used as a cancer chemotherapeutic agent. The results indicated sizes of 196 ± 5 nm and 288 ± 10 nm with a surface charge of −22 ± 3.4 mV and −17.4 ± 0.5 mV for HSA-NPs (20 mg/mL of HSA, 0.01 mg/mL of cysteine, and 10 mg/mL of urea) and HSA–HU-NPs (2 mg/mL of HU), respectively. The yield of the HSA–HU-NPs was ~93% with an encapsulation efficiency of ~77%. Thus, the particles created (immobilized with HU) were stable over time and able to prolong the effect of the drug.

## 1. Introduction

Nanoparticles or nanocapsules in the pharmaceutical sense are stable, solid colloidal particles consisting of biodegradable polymer or lipid materials, ranging in size from 10 to 1000 nm [1]. These nanoparticles attract a great deal of attention for creating drug carriers [2,3], for anticancer application. Immobilization of anticancer drugs (drug substances) in nanoparticles reduces the overall toxicity of chemotherapy, contributes to the accumulation of biologically active compounds in solid tumor tissues, and improves the solubility of drugs and pharmacokinetics [4,5]. A particular stimulus to research in this area was obtained after the Maeda and Matsumura described the enhanced permeability effect (EPR) [6,7]. EPR explains the ability of macromolecules to accumulate in the tissues of a tumor due to the relatively large pore sizes (about 200–700 nm) of the endothelium of blood vessels of a tumor in comparison with healthy tissues. Therefore, relatively large drugs and immobilized macromolecules can penetrate into the tumor cells of the endothelium avoiding healthy tissues [4].

Serum albumin plays a special role among biocompatible polymeric materials for drug nanocarriers. In medical practice, human serum albumin has been used in the treatment of shock, burns, hypoalbuminemia, as well as after surgical injuries, arthritis, etc. [8,9,10]. Albumin is a unique carrier of drugs into the target organs, as it has the ability to act as a transporter of low-molecular substances, including drugs of a various nature [1,2,8,9,10,11,12,13,14,15,16]. Examples of albumin binding of such preparations of protein origin include “Albuferon” and “Levemir” ensuring a prolonged effect [11]. The safety of the in vivo use of albumin was demonstrated by the use of the drug “Abraksan” recommended for use in the treatment of breast cancer [11].

Typically, serum albumin nanoparticles (NPs), like NPs of many natural polymers, can be synthesized by denaturing the protein in a water-in-oil emulsion and by using the coacervation method. The method of creation of NPs in an emulsion has some drawbacks that limit its use, one of which is the need to remove residual emulsifiers, stabilizers, and other organic components after manufacture. Kreuter J., Langer K. and colleagues proposed a desolvation method as an alternative for the synthesis of serum albumin NPs [12,13,14]. When albumin NPs are obtained in this way, albumin is dissolved in water, desolvated with ethanol, and then stabilized by the addition of a crosslinking agent, which is glutaraldehyde. This method is widely used by many researchers [17,18,19,20,21].

In most cases, aldehydes are used to crosslink nanoparticles or hydrogels based on albumin of various natures. In this study we attempted to synthesize albumin nanogels using exclusively natural reagents unlike the previously described known methods. In this novel approach for the production of albumin nanogels, protein molecules are first strongly denatured in the presence of urea, and then the reducing agent, cysteine, is added. Using this approach will avoid non-protein inclusions within the albumin nanomatrices. Thus, potential cytotoxic effects of the use of low molecular weight aldehydes (in particular glutaraldehyde) in the synthesis of albumin nanoparticles can be drastically reduced by employing natural agents for this purpose.

Despite the fact that the effect of urea and thiols on protein molecules was known [22], studies of their effects on the stability of albumin nanoparticles so-far were not carried out. In the present study HSA nanogels stabilized by urea and cysteine were produced, and antitumor drugs into nanoparticles were incorporated.

## 2. Materials and Methods

### 2.1. Reagents and Chemicals

Human serum albumin (lyophilized powder, 98%), and l-cysteine (98.5%) were purchased from Sigma Aldrich (Saint Louis, MO, USA). Hydroxyurea (hydroxycarbamide) was purchased from Bristol-Myers Squibb Company (New York, NY, USA). Urea (99.5%) was purchased from “HimPribor-SPb” (Saint Petersburg, Russia). Ethanol and all other chemicals were purchased from DosFarm (Almaty, Kazakhstan).

### 2.2. Process Optimization and Preparation of HSA–HU-NPs

#### 2.2.1. HSA-NPs

HSA NPs were prepared by the desolvation process. The calculated amount of HSA was dissolved in 25 mL of Milli-Q water (approximately pH 7.4) and stirred at 500 rpm at room temperature (25 °C) for 10 min. Starting HSA concentrations of 10 mg/mL, 20 mg/mL, and 30 mg/mL were prepared in 10 mL of deionized water. Subsequently, the dissolved protein was transformed into NPs by continuous addition of ethanol (1 mL/min) thus forming a turbid suspension. Calculated amounts of urea aqueous solution were added after 16 min providing concentrations of 5, 10, 20, 40 mg/mL in the HSA solution, then the l-cysteine solutions in the concentrations of 0.001 mg/mL, 0.01 mg/mL, 0.1 mg/mL, 0.3 mg/mL, and 0.5 mg/mL were added to cross-link the desolvated HSA NPs. The reaction was kept under constant magnetic stirring for 2 h. Next the NPs suspensions were purified by three cycles of centrifugation at 14,000 rpm (Eppendorf, Hamburg, Germany) for 15 min to remove non-desolvated HSA, l-cysteine, and ethanol. The NPs were redispersed in the same volume of deionized water (10 mL) for each centrifugation step by using an ultrasonication bath (Launch Tech, Shenzhen, China) for 10 min.

#### 2.2.2. HSA–HU-NPs

A pre-prepared hydroxyurea solution was added to the obtained HSA-NPs, so that the concentrations of the drug in the system were 2, 4, 6, and 8 mg/mL. Adsorption was carried out with the phosphate buffer solution (pH = 7.3) at a temperature of 25 ± 0.1 °C. The reaction was kept under constant magnetic stirring (stirring velocity 300 rpm) for 2 h. Next the NP suspension was purified by three cycles of centrifugation at 14,000 rpm (Eppendorf) for 15 min to remove not adsorbed hydroxyurea.

### 2.3. Nanoparticles Size Measurement, Zeta Potential Analysis, and Surface Morphology

The average particle size of the HSA-NPs, both with and without hydroxyurea, was measured by dynamic light scattering (DLS) using a particle size analyzer (Zetasizer NanoZS90, Malvern Instruments Limited, Worcestershire, UK). The samples were diluted with deionized water and measured at a scattering angle of 90° and a temperature of 25 °C. The polydispersity index (PI) gave an estimate of the size distribution of the HSA-NPs. The zeta potential was measured by a zeta potential analyzer (Malvern Instruments, Worcestershire, UK) using electrophoretic Laser Doppler Anemometry. The size, shape, and surface morphology of the HSA-NPs were examined by scanning electron microscopy (MIRA 3LM TESCAN, Brno, Czech Republic, EU).

### 2.4. Yield and Encapsulation Efficiency of HSA-NPs

The yield of the HSA-NPs was measured by UV-spectrophotometry. A standard curve of HSA dissolved in a solution of phosphate buffered saline (PBS) was used as a reference. The absorbance values for HSA were measured at 280 nm. The following equation was used for the calculation of the yield.

Yield [%] = (weight of HSA in solution/initial weight of HSA used) × 100.

The HSA–HU-NPs were spin-concentrated using Amicon centrifugal filters (Cedarlane, Burlington, ON, Canada) with a molecular weight cut off (MWCO) of 10,000 Da to calculate the encapsulation efficiency of hydroxyurea in HSA-NPs. This allowed the non-encapsulated hydroxyurea drug to be eluted into the collection tube. The concentration of non-encapsulated hydroxyurea was determined by the high performance liquid chromatography (HPLC) method (Shimadzu LC-20 Prominence).

### 2.5. Determination of the In Vitro HU Drug Release

A standard hydroxyurea curve was constructed to analyze the amount of unbound drug [20]. Aqueous solutions using different concentrations of the drug were prepared. The UV detector was adjusted to a wavelength of 214 nm. An Agilent 300 Extend (Agilent Technologies, Tokyo, Japan) C-18 (5 µm, 4.6 mm × 250 mm) column was used. The column temperature was settled at 40 °C. Injection volume was 10 µL. The measurement time was 10 min. The release was measured at intervals of 0, 2, 4, 8, 12, 18, and 24 h. The amount of drug that was adsorbed was calculated using the following formula:

Encapsulation efficiency (EE%) = (concentration of HU encapsulated/starting concentration of HU used) × 100.

The HU release profiles of HSA-NPs were analyzed for three different samples prepared at initial concentrations of HU 2, 4, 6 mg/mL. Samples of HSA–HU-NPs were designated as HU-2 mg/mL, HU-4 mg/mL, and HU-6 mg/mL. HSA–HU-NPs obtained under optimized conditions were dispersed in 5 mL of physiological phosphate-buffered saline (PBS) (pH = 7.4) at 200 rpm and 37 °C. The amount of HU released into the medium was determined in an interval of 24 h and compared with a control sample containing HSA-NPs without HU.

## 3. Results and Discussion

### 3.1. Nanoparticles Optimization and Preparation

Previous studies [23] concerning the preparation of spongy cryogels showed the possibility of the formation of heterophase gel systems in the presence of natural chaotropic agents in order to form a macroporous morphology. These authors repeatedly have stated that an increase in the concentration of the denaturing and reducing agents (urea and cysteine) in the initial solutions of albumin did not lead to gelation at room temperature. This can be explained by the need for cumulative effects of the denaturant and the reducing agent, as well as the effect of cryoconcentration of precursor substances in the unfrozen liquid microphase. Nevertheless, by taking into account nanoscale effects, we attempted to use these natural agents without freezing to obtain albumin nanogels.

The dependence of the formation of primary gel structures on the concentration of precursors (albumin, urea, and cysteine) was first studied to assess the degree of influence of various factors on the properties of albumin nanoparticles. In addition to assessing the polydispersity at a qualitative level for the formed albumin nanogels, the following characteristics of the efficiency of the formation of nanostructures were determined, namely the polydispersity index, zeta potential, nanoparticle yield, etc.

#### 3.1.1. Effect of HSA Concentration on Nanoparticle Size

Experimental data showed that when the initial HSA concentration was 10 mg/mL, the nanoparticle yield decreased sharply, and when the protein concentration in the initial solution was above 30 mg/mL, two or more groups of particles were formed, some of which were much larger than NP sizes. Thus, the interval from 10 to 30 mg/mL was chosen as the working range for the initial albumin concentrations. Some authors [24] showed that in order to obtain spongy cryogels gel fractions were practically not formed when the albumin concentration was below 30 mg/mL. Therefore they used more concentrated solutions for the synthesis of the cryogels. However, they did not observe the formation of gels at the nanoscale, that do not require a freezing and thawing procedure.

**Figure 1:**
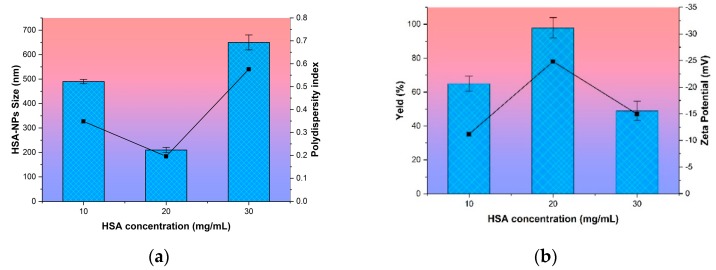
Effect of the concentration of human serum albumin on (**a**) nanoparticle size and polydispersity index of human serum albumin nanoparticles (HSA-NPs) and (**b**) the zeta potential and yield of HSA-NPs. Size and yield are represented as columns, and polydispersity index and zeta potential are represented as lines.

It follows that the most optimal initial albumin concentration in the preparation of nanoparticles is 20 mg/mL from the data presented in Figure 1.

At this concentration that the highest yield of NPs HSA (100%), the smallest average particle size (196 ± 5 nm), low PI value and a high absolute zeta potential (−22 ± 3.4 mV) was achieved. This system of the NPs with an initial HSA concentration of 20 mg/mL was relatively stable over time. An analysis of the samples within two days showed neither aggregation of particles nor destruction of the biopolymer structure. Particle size was maintained in the range of 190–210 nm.

#### 3.1.2. Effect of Urea and Cysteine Concentration on Nanoparticle Size

As expected, the addition of urea separately to ethanol-desolvated albumin particles did not stabilize them, apparently due to the fact that the urea-induced denaturation of serum albumin globules is not sufficient to form stable inter-chain hydrophobic contacts [23,24]. More efficient formation of nanogel structures was achieved only with the additional action of thiols, in this case cysteine [23,24], which is able to cleave intramolecular disulfide bonds in protein macromolecules, which leads to more complete unfolding of the polypeptide chain. This facilitates the diffusion of cysteine to the intramolecular S–S bridges inside the HSA globule. Further formation of the tertiary albumin structure is attributed [23,24] to the formation of intermolecular disulfide bonds, according to the mechanism of thiol-disulfide exchange.

Below there are results of investigation of the effect of urea and cysteine concentrations on particle size and polydispersity index (Table 1, Section I).

**Figure 2:**
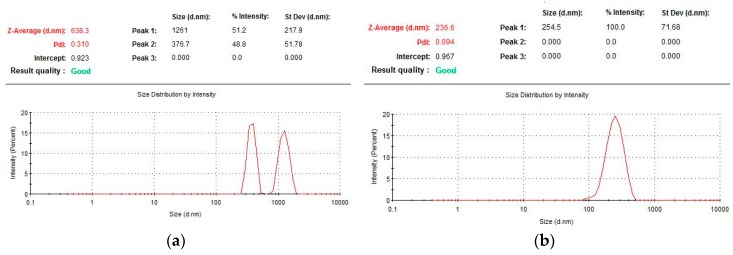
Particle size of HSA determined by photon correlation spectroscopy at various concentrations of cysteine: (**a**) 0.001 mg/mL and (**b**) 0.01 mg/mL.

Particles formed at a cysteine concentration of 0.001 mg/mL had a binary particle size distribution forming two groups, one within 700–2000 nm (average diameter of 1261 nm, the proportion of particles from the total number is 51%) and a second of 250–600 nm (average diameter of 377 nm, 49% content). It is necessary to note the non-stability of these particles, as can be seen from the control of their size (Figure 2), so, within two days, the average particle size decreased by more than three times, after four days it was impossible to detect any nanoparticles at all.

The characteristics of nanoparticles formed by varying the concentration of cysteine in the range of 0.001 to 0.5 mg/mL indicate that their optimal values are observed when the ratio of S–S bridges of the protein and the reducing agent is other than equimolar (Figure 2b). The cysteine concentration was more than an order of magnitude lower than the molar concentration of disulfide bridges in HSA. This probably is due to the participation of the thiol in protein structuring not only through the formation of covalently cross-linked biogels through thiol-disulfide exchange, but also by non-covalent association through hydrophobic interactions, resulting in expanded HSA globules in the presence of a denaturant [23,24]. Excessive addition of cysteine (above 0.01 mg/mL) leads to the restoration of the formed inter-chain S–S bridges, thus disrupting the resulting three-dimensional gel structure of albumin. As a result, the macrostructures can unfold, which can lead to the formation of larger particles and to higher values of the dispersity index (Table 1, Section I).

Another key factor that significantly influenced both the possibility of formation and the characteristics of HSA nano-gels was the concentration of urea in the initial solutions. The effect of the denaturation concentration on the formation of serum albumin NPs was studied in order to select the optimal amount of urea. As a result, the low frequency response of HSA with the above physico-chemical characteristics was obtained (Table 1, Section II). It is clear that the particle size had a pronounced this dependence on the concentration of denaturing agent from the data presented. An increase in the initial urea concentration from 5 to 10 mg/mL leads to a reduction in particle size from 380 to 310 nm. However, a further increase in the concentration of urea did not lead to a better performance, but on the contrary, the particle size of HSA increased slightly from 310 to 340 nm. A possible cause of extreme dependence may be the following. At the initial stage, urea as the denaturing agent, unfolds the biopolymer molecule and facilitates the access for cysteine molecules to interact with the protein macromolecules and then covalently links the HSA macrochain through the formation of S–S-bridges. Unfolding HSA globules in the presence of urea can also increase the hydrophobic interaction and promote non-covalent associations of protein macromolecules [23,24]. Further addition of urea above the critical concentration leads to formation of larger particles. In contrast to previous ideas about the stability, these systems are quite resistant to coagulation and/or destruction within the investigated ratios of the mixture components. The high stability of the dispersions of biopolymers allows us to count on the possibility of using low-frequency components of HSA as drug carriers without additional stabilizers.

#### 3.1.3. Effect of Hydroxyurea Addition in Nanoparticles

The antitumor drug, hydroxyurea (HU), was included into the nanoparticles of HSA as a model biologically active substance. The immobilization was carried out by adsorption HU on empty nanoparticles of HSA, that were successfully obtained using the natural cross-agents albumin, urea and cysteine. Using the above-described formulations, placebo HSA NPs with a particle diameter of 197 ± 12 nm, PI 0.102, and zeta potential (mV) −22 ± 5 were obtained. The particles were lyophilized, and their yield compared to the initial albumin concentration was 20% ± 1.5%. After repeated dissolution in deionized water, the particle size did not change significantly and was 265 ± 1.5 nm. The obtained NPs of HSA were incubated with hydroxyurea for 3 h. The concentration of the drug varied from 2–8 mg/mL. Results are presented in Table 2.

The results presented in the table above indicated a significant effect of the drug concentration on the most important characteristics of the system. The highest encapsulation efficiency was observed when the content of the drug was 6 and 8 mg/mL and 68% and 77%, respectively. The average particle size varied between 277 and 336 nm. A higher PI value was obtained for a concentration of 8 mg/mL; the PI was lower than 0.3 for other formulations. A multimodal size distribution profile was observed at a concentration of 8 mg/mL, while at a content of 2 to 6 mg/mL, a monomodal size distribution seemed to prevail. Size uniformity is an important issue in the preparation of nanoparticles, since size affects the chemical and biological properties of nanoparticles [25,26]. The formation of particles smaller than 300 nm is preferred because of the more favorable pharmacological profile, prolonged kinetics in the circulatory system, and slow release in the places of action [25,26]. The zeta potential in all cases had a negative value, however, this value was minimal when 8 mg/mL of HU was added to the initial solution. In other cases, the zeta potential ranged from −17 to −22 mV, which indicates the stability of the particles due to the repulsive forces of like charges accumulated on the surface. Considering the effectiveness of HU encapsulation in NPs, their size and zeta potential for the in vitro analysis of drug release and drug characteristics samples obtained at the initial concentration of HU in NPs HSA 2, 4, and 6 mg/mL were selected.

### 3.2. Physical-Chemical Characteristics of Nanoparticles Immobilized with HU

**Figure 3:**
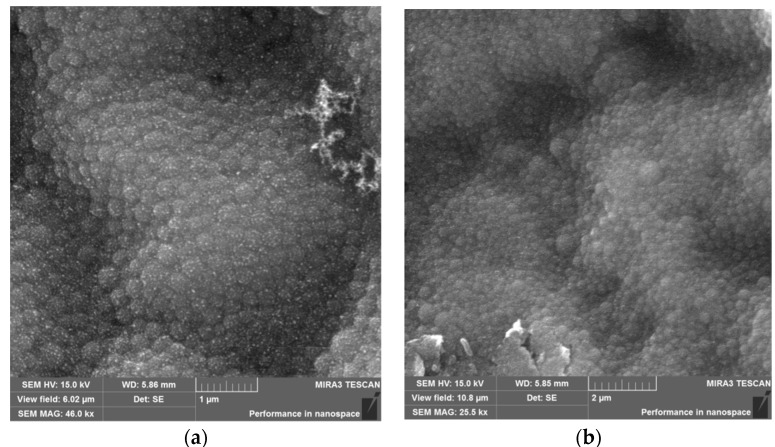
Microscope images: (**a**) Scanning electron microscope (SEM) image of HSA-NPs of a size of 196 ± 5 nm and a charge of 15 kV prepared under optimized experimental conditions (scale = 1 µm); and a (**b**) SEM image of HSA–HU-NPs, size of 288 ± 10 nm and a charge of 15 kV prepared from optimized experimental conditions with 2 mg/mL starting HU concentration (scale = 2 µm).

The morphological analysis of the NPs HSA and HSA–HU-NPs samples was performed using SEM, and the resulting images are shown in Figure 3. Both types of synthesized NPs had a spherical morphology and an average size of less than 200 nm (196 ± 5 nm and 288 ± 10 nm, respectively).

**Figure 4:**
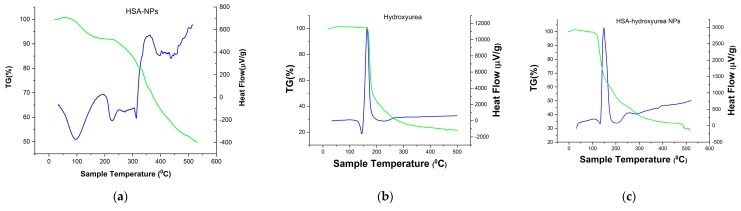
Thermogravimetric curves of (**a**) HSA-NPs; (**b**) HU; and (**c**) HSA–HU-NPs.

A thermogravimetric (TG) analysis (differential scanning calorimetry (DSC)) of the individual system components and the resulting NPs was performed to confirm the inclusion of HU in the albumin NPs complex. Figure 4 shows the TG–DSC (thermogravimetric differential scanning calorimetry) curves of hydroxyurea, HSA-NPs and HSA nanoparticles loaded with HU. For hydroxyurea, a characteristic endothermic peak was observed at 145 °C with a change in enthalpy (Figure 4b), which was not accompanied by a mass loss, and characterized the HU phase transition (a value close to the melting point). Further, an exothermic peak was observed at a temperature of 168 °C, there was a mass loss of up to 53% in the range of 150–180 °C, which might correspond to the decomposition temperature of the drug [27,28]. HSA-NPs exhibited an endothermic peak at 96 °C (Figure 4c), which probably corresponded to the period of their melting. However, a significant shift of temperature to the region of large values was observed when compared with the pure albumin DSC curves [29]. Perhaps this was due to the greater structuring of albumin in the NP. Further, when the HSA–HU-NPs samples were heated, another endothermic peak was observed by DSC at a temperature of 230 °C, which completely coincided with the albumin not structured into nanoparticles. The next peak on the DSC curve corresponded to the decomposition of the NP protein at a temperature of 313–351 °C with a weight loss of more than 50%. The DSC curve for HU immobilized into nanoparticles (Figure 4c) presented the peaks characteristics of the HU but showing lower intensity peaks. This thermal behavior suggests that a drug did not change the chemical nature as a result of immobilizing albumin in the NP, this information corroborated with FT-IR results.

**Figure 5:**
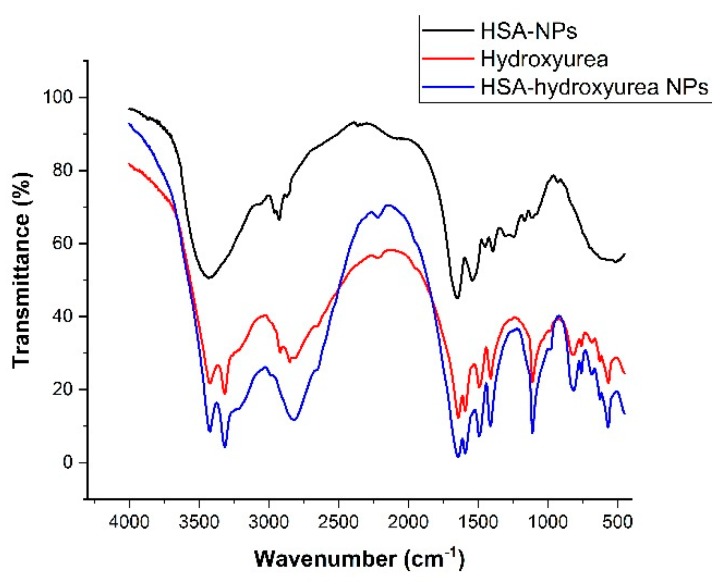
FT-IR spectra of hydroxyurea, HSA NPs, and hydroxyurea-loaded HSA NPs.

Samples of HSA NPs, HU, and HSA–HU-NPs were examined using an FT-IR spectrophotometer. This technique was used to assess chemical and conformational changes that occur when NPs are formed or when they interact with other compounds through a slight shift in the characteristic bands in the spectral regions of amide A and amide B [29,30]. Figure 5 shows the spectra of pure drug and albumin, as well as adsorbed nanoparticles.

In Figure 5 the main bands of pure albumin at 3284 cm^−1^ (amide A bound to NH), 2960 (amide B bound to the free ion), 1644 cm^−1^ (amide 1, C=O bond), 1535 cm^−1^ (amide II associated with CN stretching vibrations and bending vibrations of NH), 1393 cm^−1^ (CH_2_ bending groups), and ~1245 cm^−1^ (amide III associated with C–N stretching vibrations and bending vibrations of N–H) can be seen. When compared with the peaks of the HSA–HU-NPs and the initial HSA-NPs, small shifts of 2–3 nm were observed, which may indicate the structuring of the nanoparticles and the formation of associated bonds. Peaks at 3422 cm^−1^ (belonging to antisymmetric stretching of NH_2_), 3316 cm^−1^ (referring to NH), 3220 (NH_2_ symmetric vibrations), 1644 cm^−1^ (amide 1, C=O bond), 1594 cm^−1^ (deformation vibrations of amide 2), and 1493 cm^−1^ (deformation vibrations of NH) are characteristic for hydroxyurea. The spectrum of HSA–HU-NPs shows characteristic peaks of the protein and the structure of hydroxyurea, which indicates the absence of chemical interaction between HSA-NPs and hydroxyurea. In addition, some bands showed differences in intensity (Figure 5), for example, the intensities in the amide B and amide III bands are reduced, which may indicate the formation of non-chemical bonds between protein molecules and hydroxyurea with the participation of these groups.

### 3.3. In Vitro Drug Release

**Figure 6:**
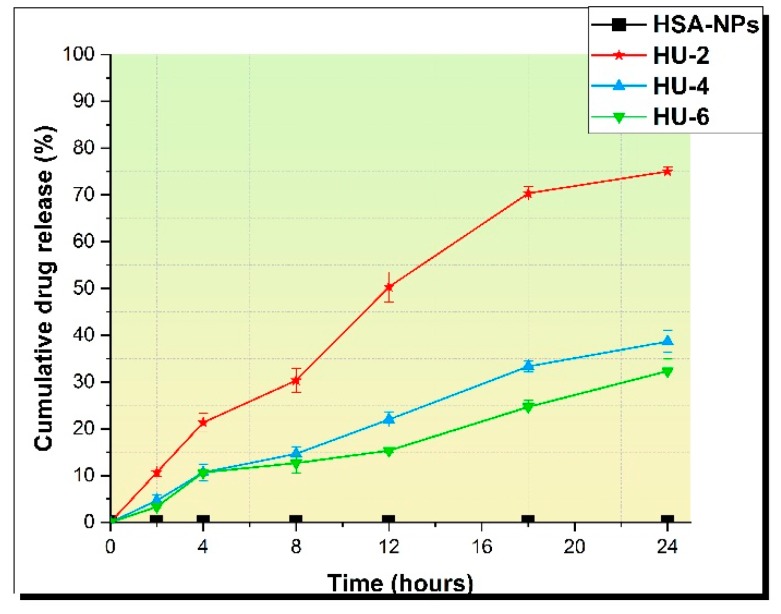
Drug release during 24 h.

The HU release profiles of HSA-NPs were analyzed for three different samples prepared at initial concentrations of HU 2, 4, and 6 mg/mL. Samples of HSA–HU-NPs were designated as HU-2 mg/mL, HU-4 mg/mL, and HU-6 mg/mL. HSA–HU-NPs obtained under optimized conditions were dispersed in 5 mL of physiological phosphate-buffered saline (PBS; pH = 7.4) at 200 rpm and 37 °C. The amount of HU released into the medium was determined in an interval of 24 h and compared with a control sample containing HSA-NP without HU as shown in Figure 6. Approximately 76% ± 5.0%, 38% ± 7%, and 32% ± 5% of the drug were released within 24 h from HSA–HU-NPs prepared at HU concentrations of 2 mg/mL, 4 mg/mL, and 6 mg/mL, respectively. The drug release became much slower after 24 h for all three samples. About 50% ± 5% of the drug was released within 12 h with sample HU-2 mg/mL. Later, the total yield of the drug release reached 76% ± 5.0% within 24 h. For samples HU-4 mg/mL and HU-6 mg/mL, the drug was released more slowly, i.e. 38% ± 7% and 32% ± 5% of HU over 24 h, respectively. These data are comparable with the results of the study with the release of drugs in vitro using similar biologically active substances with bovine serum albumin nanoparticles and HSA-NPs [23]. This allows for a continuous affect on the cancer cells achieving a prolonged action regarding the decrease in cell viability over time in contrast to a full release of the drug within 12 h.

## 4. Conclusions

HSA nanoparticles are promising for the transport of drugs within the body. Traditionally, HSA nanoparticles can be synthesized by desolvation of an aqueous solution of HSA in ethanol with subsequent stabilization using a crosslinking agent, for which glutaraldehyde is used. The method presented here allows the elimination of the use of a synthetic stabilizer by replacing it with natural agents. Urea and cysteine were tested in the present study as such natural agents. Urea as a denaturing agent that unfolds the biopolymer molecule and facilitates the access for cysteine molecules to interact with the protein molecules. The effectiveness of the formation of nanostructures is influenced by various factors including precursor concentration (urea, albumin, and cysteine). The optimal values for particle size, polydispersity index, zeta potential, and particle yield were achieved at the albumin concentration of 20 mg/mL, cysteine of 0.01 mg/mL, and urea of 10 mg/mL. By variation of these parameters, a particle size of less than 200 nm was achieved. The addition of hydroxyurea to the HSA nanoparticles solution formed the HSA–HU-NPs complex. Particles with satisfactory physico-chemical parameters were formed at an initial concentration of hydroxyurea ranging from 2–6 mg/mL. In this case, the loading efficiency was 40%–60% with a particle size of 277–288 nm. Morphological analysis of the samples showed that HSA–HU nanoparticles were spherical and uniform in size. Thermal and spectral studies suggest that the drug did not change the chemical nature as a result of immobilization into the polymer matrix. In vitro studies showed the possibility of creating a prolonged antitumor drug release and suggest effectiveness in the next stages of this project. Our future studies will include an in vitro cytotoxicity study for cancer cells. We will also work on the creation of a system for the targeted delivery of the complex HSA–HU-NPs.

## Figures and Tables

**Table 1:** The effect of cysteine concentration on the physico-chemical characteristics of empty albumin NPs: [HSA] = 10 mg/mL, T = 25 °C.

Precursor Concentration		Particle Characteristics					
		Immediately after Purification		After 2 Days		After 4 Days	
		Average Diameter NPs, nm	PI	Average Diameter NPs, nm	PI	Average Diameter NPs, nm	PI
Urea, mg/mL	Cysteine, mg/mL						
**I**							
40	0.001	308 ± 40	0.350	112 ± 6	0.572	-	-
	0.01	237 ± 1.5	0.094	240 ± 4	0.062	264 ± 5	0.084
	0.1	285 ± 2	0.073	304 ± 2	0.053	299 ± 8	0.188
	0.3	321 ± 2.5	0.114	409 ± 6	0.085	429 ± 12	0.066
	0.5	331 ± 3	0.116	355 ± 2	0.158	386 ± 6	0.057
**II**							
5	0.5	378 ± 15	0.115	389 ± 12	0.304	375 ± 14	0.540
10		310 ± 5	0.085	320 ± 7	0.058	317 ± 5	0.092
20		389 ± 7	0.114	392 ± 8	0.176	388 ± 9	0.165
40		398 ± 10	0.128	385 ± 9	0.125	399 ± 12	0.105

**Table 2:** Characteristics of HSA nanoparticles containing hydroxyurea.

The Concentration of HU mg/mL	Particle Size (nm)	Encapsulation Efficiency	PI	Zeta Potential (mV)	Size Distribution
2	288 ± 10	40 ± 2	0.15	−17.4 ± 0.5	100% (210–454)
4	336 ± 2.5	22 ± 0.5	0.24	−16.6 ± 0.4	100% (205–556)
6	277 ± 1.5	68 ± 2	0.20	−22.5 ± 0.7	100% (170–425)
8	312 ± 2	77 ± 4	0.59	−3.3 ± 0.2	45% (720–1265); 29% (177–550); 26% (32–90).
